# Supplementary material for: Palliative Gastrectomy Improves the Survival of Patients with Metastatic Early-Onset Gastric Cancer: A Retrospective Cohort Study
Source: Curr Oncol. 2023 Aug 27;30(9):7874–90. doi: 10.3390/curroncol30090572 (PMC10527682; doi:10.3390/curroncol30090572)
Supplement: Supplementary file 1 [file curroncol-30-00572-s001.zip › Supplemental Table S1 Cox_cancer_cause.pdf]

Supplemental Table S1: Prognostic factors for cause- specific survival

Variable	Univariable Cox regression analysis [a]			Multivariable Cox regression analysis [b]		
	Hazard ratio	95% CI	P value	Hazard ratio	95% CI	P value
Age, year	1.001	0.988-1.015	0.862			
Male sex	0.935	0.779-1.121	0.465			
Race						
White	reference					
Black	0.976	0.755-1.262	0.853			
American Indian/Alaska Native	1.485	0.702-3.141	0.301			
Asian or Pacific Islander	0.841	0.662-1.069	0.157			
Unknown	0.426	0.106-1.715	0.230			
Marital status						
Married	reference					
Single	0.869	0.707-1.069	0.185			
Divorced/Widowed/Separated	0.858	0.632-1.165	0.326			
Unknown	1.016	0.604-1.710	0.951			
Year of diagnosis	0.966	0.945-0.987	**0.002**	0.974	0.932-1.017	0.232
Primary site						
Cardia	reference			reference		
Non- cardia	1.412	1.097-1.817	**0.007**	1.323	1.006-1.740	**0.045**
Overlapping lesion of stomach	1.491	1.074-2.072	**0.017**	1.360	0.960-1.926	0.084
Unknown	1.606	1.176-2.193	**0.003**	1.455	1.044-2.026	**0.027**
Tumor differentiation grade						
I/II	reference					
III/IV	1.309	0.993-1.726	0.056			
Unknown	1.080	0.738-1.580	0.692			
Histology						
Signet ring cell carcinoma	reference			reference		
Other adenocarcinoma	0.810	0.674-0.974	**0.025**	0.848	0.697-1.031	0.097
Non- adenocarcinoma	0.608	0.384-0.963	**0.034**	0.585	0.359-0.951	**0.030**
Tumor size						
≤5 cm	reference					
>5 cm	1.012	0.814-1.258	0.915			
Unknown	1.120	0.894-1.404	0.323			
T stage						
T1/ T2	reference					
T3/ T4	1.310	0.922-1.861	0.131			
Tx	1.319	0.821-2.118	0.252			
Metastasis to the liver [b]						
No	reference			reference		
Yes	1.174	0.835-1.651	0.355	1.158	0.801-1.674	0.436
Unknown	1.357	1.122-1.643	**0.002**	0.846	0.088-8.108	0.884
Metastasis to the lung [b]	reference			reference		
No	2.024	1.071-3.823	**0.030**	3.308	1.669-6.557	**0.001**
Yes	1.359	1.133-1.630	**0.001**	2.393	0.334-17.134	0.385
Unknown						
Metastasis to the bone [b]						
No	reference					
Yes	1.607	0.898-2.878	0.110	1.750	0.962-3.181	0.067
Unknown	1.345	1.121-1.613	**0.001**	0.528	0.160-1.743	0.295
Metastasis to the brain [b]						
No	reference					

Yes	4.836	1.538-15.208	**0.007**	4.369	1.36-14.036	**0.013**
Unknown	1.332	1.112-1.594	**0.002**	--	--	--
Receipt of surgery						
No	reference			reference		
Yes	0.492	0.411-0.590	**<0.001**	0.462	0.383-0.558	**<0.001**
Receipt of radiation						
No	reference					
Yes	0.930	0.758-1.142	0.491			
Receipt of chemotherapy						
No	reference			reference		
Yes	0.719	0.58-0.891	**0.003**	0.655	0.522-0.823	**<0.001**

CI= confidence interval. P values in bold indicate <0.05 and are considered as statistically significant. [a] 16 patients died of unknown causes are excluded in this analysis. [b] Confounding factors (including year of diagnosis, primary site, tumor differentiation grade, histology, site of metastasis, receipt of surgery, receipt of chemotherapy) were adjusted in multivariable Cox proportional hazards regression analysis. [c] The site of metastasis was only accessible for patients diagnosed after 2010, while the site of metastasis of patients diagnosed before 2010 was recorded as unknown.
