# Supplementary material for: Palliative Gastrectomy Improves the Survival of Patients with Metastatic Early-Onset Gastric Cancer: A Retrospective Cohort Study
Source: Curr Oncol. 2023 Aug 27;30(9):7874–90. doi: 10.3390/curroncol30090572 (PMC10527682; doi:10.3390/curroncol30090572)
Supplement: Supplementary file 1 [file curroncol-30-00572-s001.zip › Supplemental Table S2 The effect of treatment modalities on overall survival and cancer-specific survival.pdf]

Supplemental Table S2: The effect of treatment modalities on overall survival and cause-specific survival.

Treatment modalities	Overall survival [a, b]			Cause-specific survival [c, d]		
	Number	Hazard ratio (95% CI)	P value	Number	Hazard ratio (95% CI)	P value
No therapy (reference)	47	reference		47	reference	
Surgery	66	0.192(0.127-0.290)	**<0.001**	63	0.193(0.126-0.295)	**<0.001**
Radiation	13	0.897(0.450-1.784)	0.756	13	0.718(0.367-1.406)	0.334
Chemotherapy	182	0.371(0.264-0.521)	**<0.001**	175	0.380(0.269-0.536)	**<0.001**
Surgery + radiation	7	0.292(0.128-0.668)	0.004	7	0.292(0.127-0.671)	**0.004**
Surgery + chemotherapy	162	0.192(0.134-0.274)	**<0.001**	159	0.194(0.135-0.280)	**<0.001**
Chemoradiation	56	0.282(0.183-0.436)	**<0.001**	54	0.298(0.192-0.464)	**<0.001**
Trimodality	63	0.207(0.137-0.315)	**<0.001**	62	0.218(0.143-0.332)	**<0.001**

P values in bold indicate <0.05. [a] Confounding factors (including year of diagnosis, primary site, histology, T stage, metastasis to the liver, metastasis to the lung, metastasis to the bone, metastasis to the brain) were adjusted in multivariable Cox proportional hazards regression analysis to evaluate the hazard ratio of treatment modalities. [b] No patients are excluded, and the total number is 596. [c] Confounding factors (including year of diagnosis, primary site, histology, metastasis to the liver, metastasis to the lung, metastasis to the bone, metastasis to the brain) were adjusted in multivariable Cox proportional hazards regression analysis to evaluate the hazard ratio of treatment modalities. [d] 16 patients died of unknown causes are excluded and the total number is 580.
