# Supplementary figures and images for: Palliative Gastrectomy Improves the Survival of Patients with Metastatic Early-Onset Gastric Cancer: A Retrospective Cohort Study
Source: Curr Oncol. 2023 Aug 27;30(9):7874–90. doi: 10.3390/curroncol30090572 (PMC10527682; doi:10.3390/curroncol30090572)

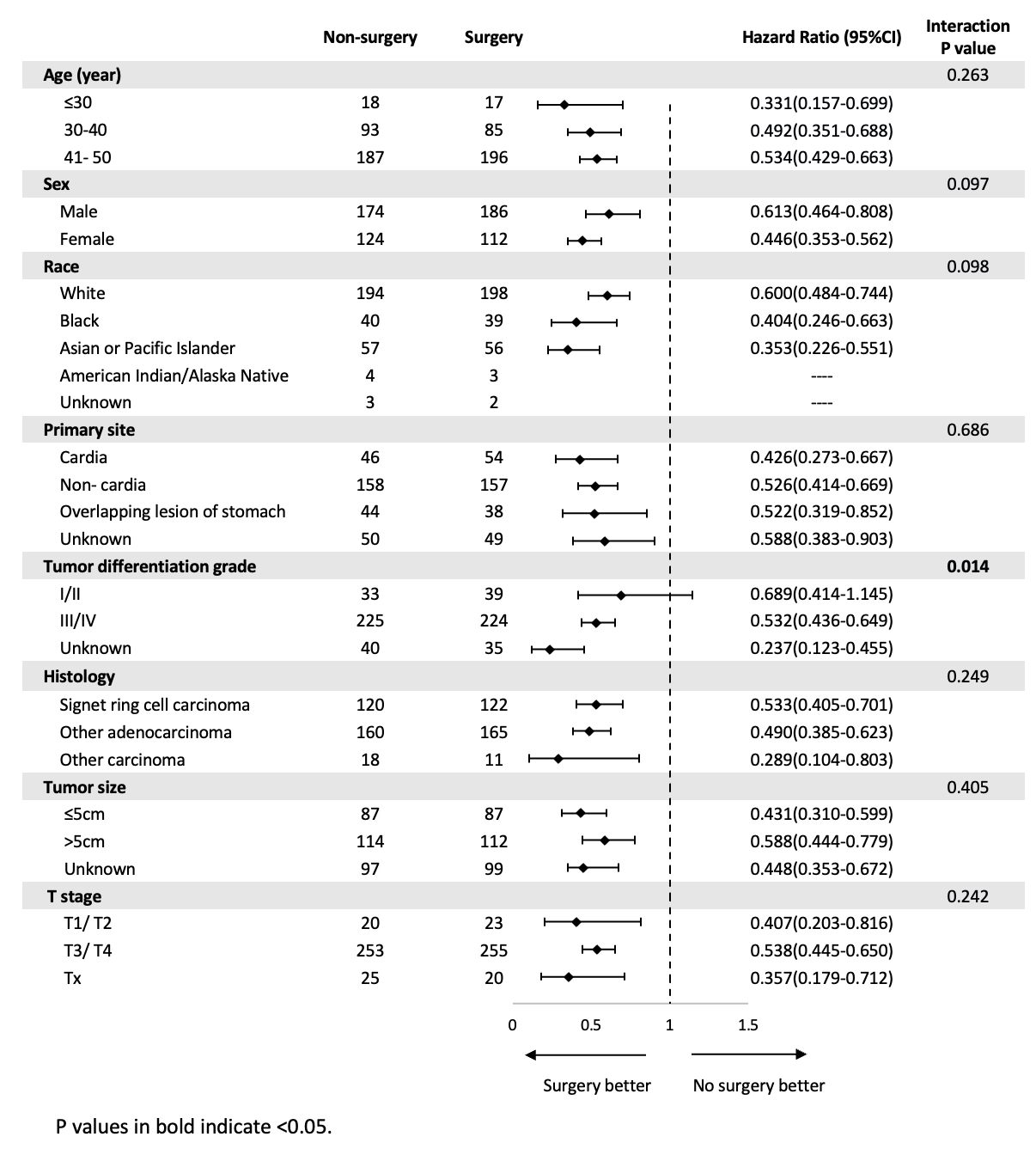

Supplement: Supplementary file 1 [file curroncol-30-00572-s001.zip › Supplemental figure S1.png]

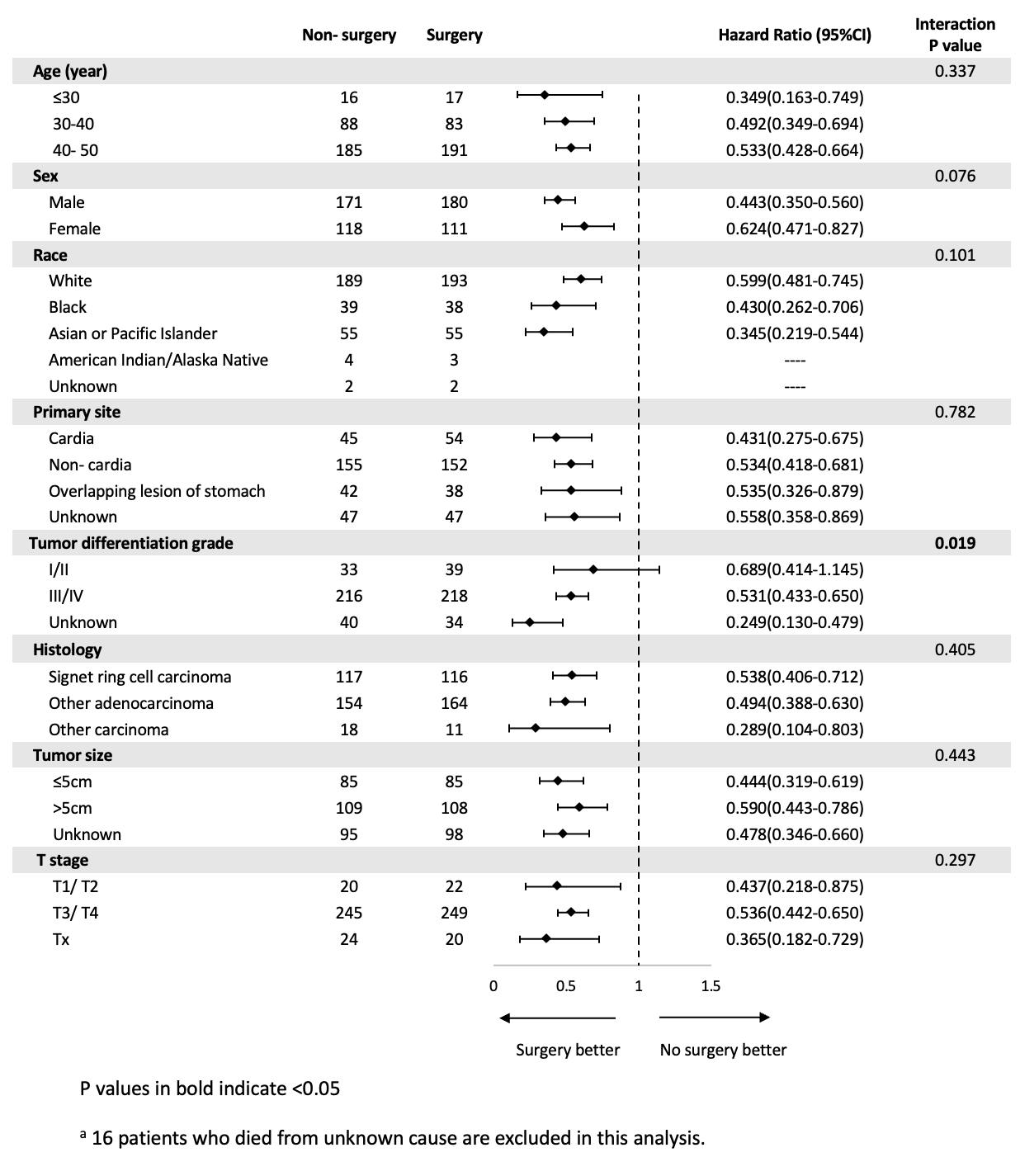

Supplement: Supplementary file 1 [file curroncol-30-00572-s001.zip › Supplemental figure S2.png]
